# Starvation-Associated Genome Restructuring Can Lead to Reproductive Isolation in Yeast

**DOI:** 10.1371/journal.pone.0066414

**Published:** 2013-07-24

**Authors:** Evgueny Kroll, Scott Coyle, Barbara Dunn, Gregory Koniges, Anthony Aragon, Jeremy Edwards, Frank Rosenzweig

**Affiliations:** 1 Division of Biological Sciences, University of Montana, Missoula, Montana, United States of America; 2 Department of Cellular and Molecular Pharmacology, University of California San Francisco, San Francisco, California, United States of America; 3 Department of Genetics, Stanford University School of Medicine, Stanford, California, United States of America; 4 Molecular Genetics and Microbiology, University of New Mexico, Albuquerque, New Mexico, United States of America; University of Arkansas, United States of America

## Abstract

Knowledge of the mechanisms that lead to reproductive isolation is essential for understanding population structure and speciation. While several models have been advanced to explain post-mating reproductive isolation, experimental data supporting most are indirect. Laboratory investigations of this phenomenon are typically carried out under benign conditions, which result in low rates of genetic change unlikely to initiate reproductive isolation. Previously, we described an experimental system using the yeast *Saccharomyces cerevisiae* where starvation served as a proxy to any stress that decreases reproduction and/or survivorship. We showed that novel lineages with restructured genomes quickly emerged in starved populations, and that these survivors were more fit than their ancestors when re-starved. Here we show that certain yeast lineages that survive starvation have become reproductively isolated from their ancestor. We further demonstrate that reproductive isolation arises from genomic rearrangements, whose frequency in starving yeast is several orders of magnitude greater than an unstarved control. By contrast, the frequency of point mutations is less than 2-fold greater. In a particular case, we observe that a starved lineage becomes reproductively isolated as a direct result of the stress-related accumulation of a single chromosome. We recapitulate this result by demonstrating that introducing an extra copy of one or several chromosomes into naïve, i.e. unstarved, yeast significantly diminishes their fertility. This type of reproductive barrier, whether arising spontaneously or via genetic manipulation, can be removed by making a lineage euploid for the altered chromosomes. Our model provides direct genetic evidence that reproductive isolation can arise frequently in stressed populations via genome restructuring without the precondition of geographic isolation.

## Introduction

Large-scale genome restructuring can occur when cells undergo stress due to environmental change [1], [2], and novel genome structures can play important roles in adaptive evolution [3]–[9], aging [10] and human disease [11]. Large scale genome restructuring also frequently accompanies speciation, notably that caused by hybridization [12]–[16], which occurs in every eukaryotic Kingdom [17] and accounts for a large fraction of extant plant species [18].

Genomic rearrangements induce synaptic aberrations in meiosis [18]–[20]. In animals and fungi, pachytene and meiotic spindle checkpoints reduce the frequency of unbalanced gametes by aborting aberrant meiotic events [21]–[23]. Because unbalanced gametes are rare and/or frequently inviable [24], [25], mechanisms that evolved to reduce chromosomal irregularities in meiosis may also act in speciation by restricting gene flow between newly-arising chromosomal variants [26]–[28]. In heterogonic taxa such as *Saccharomyces*, if an unbalanced chromosomal variant remains fertile when selfed or interbred, a nascent species may emerge.

In yeast, fertility can be viewed as the product of sporulation frequency (the ratio of sporulated cells, or asci, to the total number of cells) and spore viability (the ratio of germinated spores to total spore output). Any genetic change that drastically reduces the output of viable gametes essentially acts as a post-zygotic reproductive isolating mechanism. Indeed, biological species in the Ascomycetes are defined by very low fertility following interspecific crosses [28]; generally only spore viability is considered, owing to the fact that sporulation frequency varies within and between closely related species because of strain-specific differences in sporulation conditions [24], [25]. Nevertheless, because sporulation frequency and spore viability both ultimately determine yeast's gametic output, an experimental model of speciation by post-zygotic reproductive isolation requires both parameters to be estimated in a common genetic background.

Reproductive isolation has been difficult to reproduce experimentally in the lab, in part because conditions there are typically benign, resulting in low and fairly constant mutation/rearrangement rates [29] unlikely to lead to rapid reproductive isolation [30]. However, populations in nature frequently encounter stressful conditions that lower their mean population fitness [31] and increase the rate at which both fine-scale mutations and large-scale genome rearrangements occur [9] (and references therein). Given these observations, we decided to model the effects of stress on reproductive isolation by inducing starvation in yeast populations and determining whether barriers to gene flow arose among the survivors.

In previous experiments we showed that in yeast, a 30-day starvation treatment resulted in widespread genome rearrangements that conferred an adaptive advantage when survivors were re-starved [9]. Here, we extend our observations, by asking whether starvation-associated genome rearrangements also disrupt gene flow between lineages that survive starvation. We employed the genetically-tractable, diploid strain BY4743, an S288c derivative [32]; this strain, while sporulating less frequently than other laboratory yeast strains, has the important advantage of not spontaneously sporulating when starved. We subjected this diploid yeast to a 30-day starvation regimen and asked, at what frequency does starvation-associated genome restructuring occur relative to small-scale mutations, and what impact does genome restructuring have on fertility and species integrity? Whole genome sequencing and array-based comparative genomic hybridization indicate that the major genetic differences between an unstarved ancestor and its starvation-resistant descendants are chromosomal rather than genic. Genetic crosses and reconstruction experiments reveal that in a particular case, evolution leading to the presence of a single extra chromosome prevents meiosis, quickly and effectively creating a barrier to gene flow.

Our model provides experimental evidence that reproductive barriers can arise quickly within a population via simple chromosomal changes caused by stress, without the preconditions of geographic separation or divergent selection.

## Results

**Figure 1:**
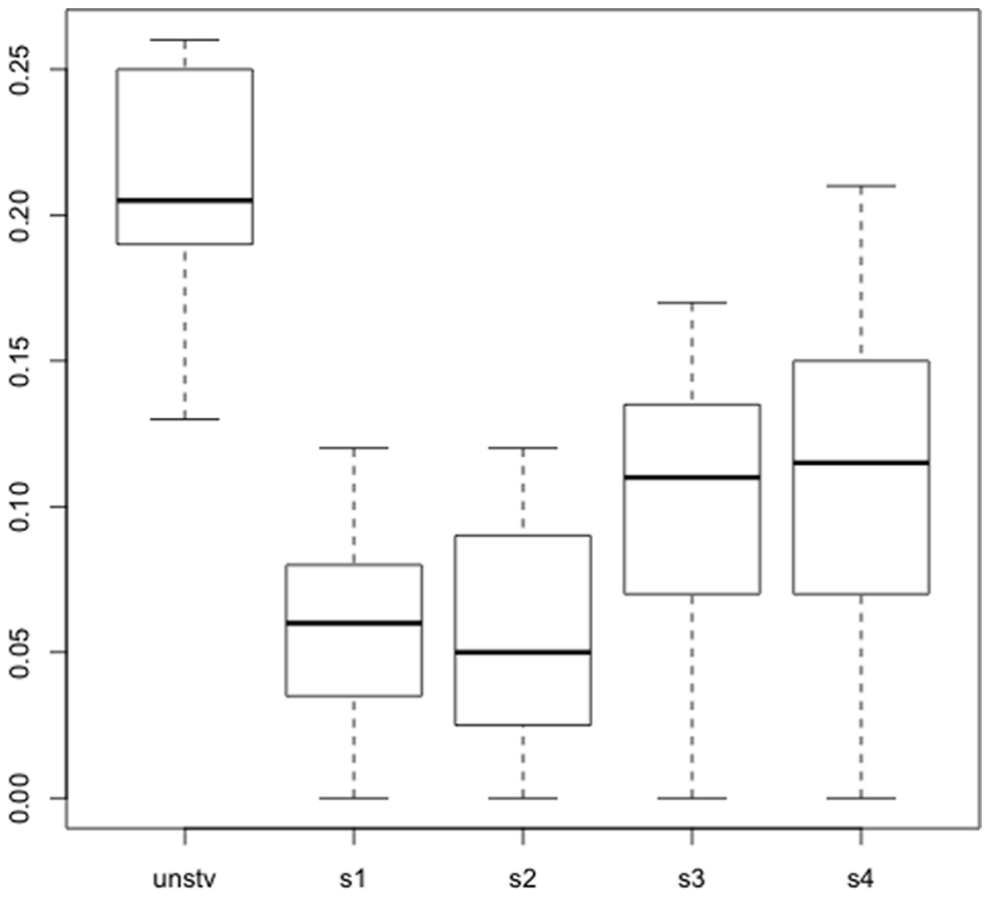
Sporulation frequencies of starved and unstarved isolates. Boxplot of sporulation frequencies for the sample of 20 isolates from each of the four starved cultures (s1, s2, s3, s4) and unstarved control (unstv). Boxes denote 50% of the data in the middle (interquartile range), central bar is the median, error bars extend to the full range of sporulation frequency values in each sample. Text provides details for the statistical treatment of the data.

To study post-starvation fertility we examined survivors from 4 parallel cultures of diploid yeast derived from a single clone, that had been starved for 1 month (*starved cultures*), as described previously [9]. By the end of this month-long treatment cells had undergone, on average, approximately 10 generations and retained approximately 50% viability. No sporulation was detected in starved cultures examined by microscopy. For comparison, our *unstarved control culture* was an overnight liquid YEPD batch culture of BY4743. Following starvation, the four starved cultures were incubated in fresh rich medium overnight, to restore their viability, then plated onto YEPD solid medium. 20 colonies from each of the four starved cultures were isolated; these starved isolates, along with similarly-isolated, unstarved control isolates, were sporulated, and their sporulation frequencies and spore viabilities recorded (**Fig. 1**).

Sporulation frequencies of the starved cultures were significantly lower than that of the unstarved control (median for the colonies from pooled unstarved control: 21%; from starved cultures: 6%, 5%, 11%, 11%, p = 0.0023, Kruskal-Wallis test), but were not significantly different among starved cultures (**Fig. 1**). In contrast, spore viability in starved cultures and unstarved control cultures ranged from 75% to 95% and was not statistically different (p = 0.25, Kruskal-Wallis test). While these findings stand in contrast with prior observations on fertility among *Saccharomyces* genus hybrids [28], they agree with the only other published study in yeast experimental speciation [33]. We further consider the significance of these findings in our Discussion.

The fact that starved cultures exhibited significantly lower sporulation frequency than the unstarved control indicated that accumulated changes in the survivors' genomes reduced their overall fertility. This suggests that spores derived from starved cells might be wholly or partially reproductively isolated from the unstarved ancestor.

### Forward mutation during starvation

Starvation can favor the accumulation of point and other small-scale mutations. Indeed, increased incidence of starvation-associated mutations has been reported in both bacteria [34]–[37] and yeast [38]. Such mutations can influence fertility by three distinct mechanisms: by mutations in sporulation genes, by mutations having negative epistatic effects (in “speciation genes”) [39]–[41], and by producing enough sequence difference to activate anti-recombinagenic mismatch repair [42]–[44].

To evaluate fine-scale mutations' contribution to sporulation defects, we measured the frequency of forward mutation to cycloheximide resistance in starved survivors. Resistance to cycloheximide mostly arises via recessive mutations at *CYH2* locus [45], and two mutations are necessary for cycloheximide resistance in a diploid strain. 5×10^7^ cells from each of the four starved cultures and the four parallel samples from unstarved population of BY4743 were plated on rich medium containing cycloheximide. After one week, cycloheximide-resistant colonies were counted and mutation frequencies, corrected for starved cultures' viability, were determined. The difference in median frequencies was 1.6-fold and not significant (four unstarved samples mutant median frequency (mmf) = 5×10^−8^, four starved cultures, mmf = 8×10^−8^; Mann Whitney *U* test, p = 0.69).

Because fine-scale mutation frequencies in starved populations were only marginally higher than those observed in unstarved populations, we concluded that they were insufficient to account for the appearance of a reproductive barrier in our experiments. With this, we note that estimating the rate of starvation-associated mutation poses special challenges. First, mutation rate estimates based on fluctuation analysis require nutrient-sufficient conditions. Second, mutant frequencies arising in prolonged “stationary-phase” populations may poorly reflect true underlying mutation rates; for example, partial clonal expansions may occur [38] that would tend to decrease the apparent mutation frequency.

### Starved cultures exhibit significantly higher rearrangement frequency

While forward mutation assays revealed low levels of fine-scale mutation in starved cultures, we did detect a substantial increase in the incidence of large-scale genomic rearrangements (GCR). Of 271 starved BY4743 diploid isolates analyzed by pulsed-field gel electrophoresis (PFGE), 18 contained novel bands (17 – a single novel band and 1 – two new bands), giving overall a 6.6% frequency of new chromosomal variants. By contrast, none of the 139 isolates from unstarved cultures exhibited chromosomal rearrangements using PFGE. This provides non-overlapping binomial 95% confidence intervals of 4.0–9.8% for rearrangement frequency in starved isolates and 0–2.3% in the non-starved control. Note that PFGE analysis of chromosomal rearrangements is conservative, as inversions and balanced translocations cannot be detected by this method. Thus the true frequency of genomic rearrangements could be higher than that estimated by PFGE.

These data show that starved yeast cultures contain an incidence of chromosomal rearrangements that is several orders of magnitude higher than what has been estimated for a typical laboratory yeast strain [46] and for the non-starved control (p = 0.0014, Fisher's exact test). These rearrangements could not have been pre-existing and simply selected during starvation, as only one-third of subclones containing rearranged chromosomes exhibited any selective advantage during starvation [9]. Also, because the spectra of rearrangements in parallel cultures were different, it is unlikely that they existed in the population prior to starvation (see also [9]).

Altogether, our data show that starved yeast cultures are characterized by a significantly increased rearrangement frequency relative to unstarved controls, while the starved cultures' fine-scale mutation frequencies do not differ significantly from unstarved controls.

### A subset of meiotic isolates sporulate poorly when backcrossed to their common ancestor

To see whether genomic changes that accumulated during starvation could create a reproductive barrier, we performed backcross analysis. We sporulated starved yeast cultures *en masse*, and randomly isolated 17 viable haploid spores (four each from the first three cultures and 5 from the fourth culture), which we termed *starved (meiotic) isolates*, germinated them and determined their mating type and auxotrophies. We then crossed the starved meiotic isolates to the unstarved haploid ancestor strains BY4741 or BY4742 (*backcross*); we also created homozygous diploids of the starved isolates by self-mating via plasmid-mediated *HO* expression (*self-cross*). We reasoned that if starved isolates were reproductively isolated from their unstarved ancestor they would exhibit significantly lower fertility in *backcross* than in *self-cross*. Sporulation frequency was used as a proxy for fertility, since spore viability was generally high (75–95%) and indistinguishable from that of spores derived from the unstarved diploid control.

**Figure 2:**
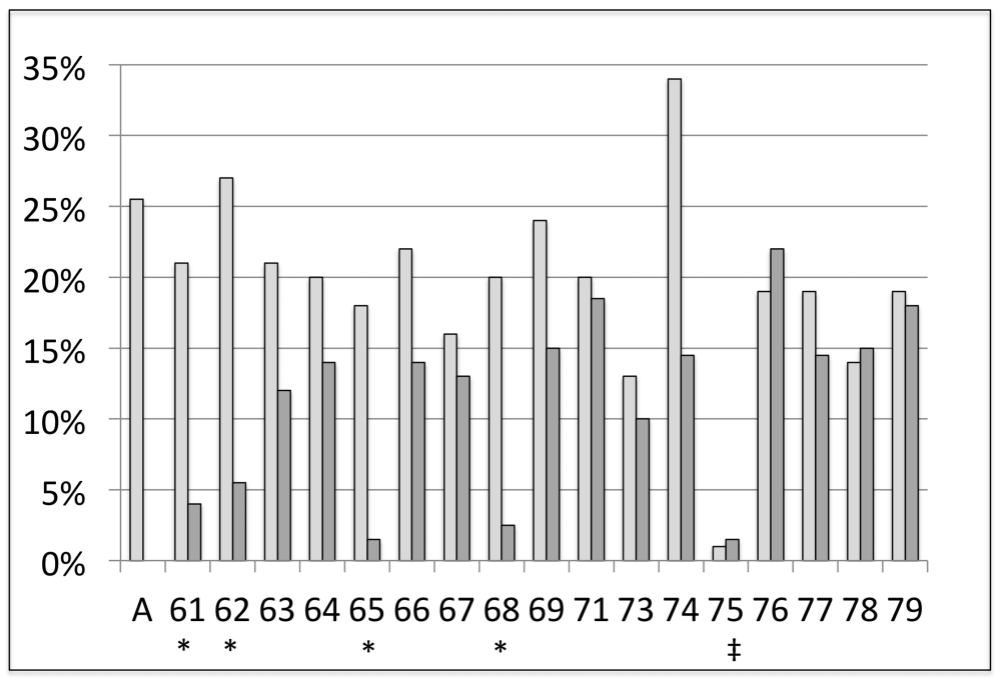
Sporulation frequencies of backcrosses and self-crosses. Crosses were made using haploid derivatives of starved isolates from four starved cultures. A – unstarved diploid control. Light grey bars are self crosses, dark grey bars are backcrosses. “*” denote significant differences between the corresponding self-cross and backcross sporulation frequencies (Bonferroni-corrected (n = 17), two-tailed Fisher's exact test at 95% confidence). “‡” denotes isolate (75a) whose self-cross lost the ability to sporulate. Sporulation frequencies among unstarved isolates backcrossed to the ancestor were indistinguishable from the diploid ancestor's (data not shown).

Using a Bonferroni-corrected Fisher's exact test at 95% confidence, we compared sporulation frequency of starved isolates in backcross and in self-cross with those of the unstarved diploid control BY4743; we also compared these values to sporulation frequency of unstarved meiotic progeny backcrossed to BY4743. We found that while one isolate (75) had low sporulation frequency in *both* backcross and self-cross (and was thus unlikely to produce meiotically competent offspring) four out of 17 starved isolates (61, 62, 65, 68 two from culture 1 and two from culture 2) exhibited significantly lower sporulation frequency in backcross than the unstarved diploid BY4743, but were fertile in self-cross (**Fig. 2**); these isolates were retained for further analysis.

### Meiotic isolate 62 was disomic for Chromosome I

**Figure 3:**
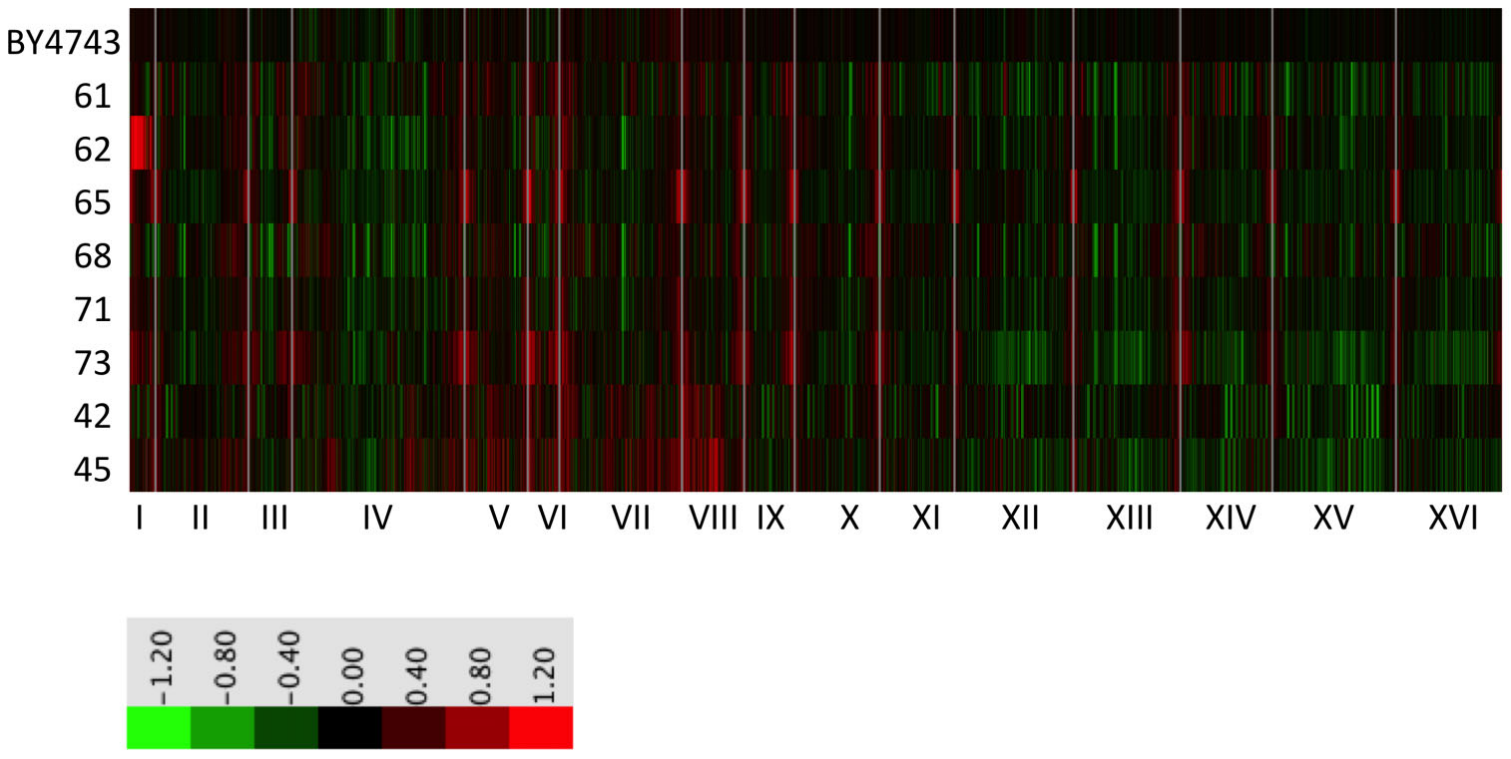
Array-Comparative Genome Hybridization of starved isolates and their shared ancestor. aCGH of the ancestral diploid BY4743, four starved isolates displaying lower fertility in backcross (61, 62, 65, 68), two starved isolates with high fertility in backcross (71, 73), and two unstarved isolates (42 and 45). Roman numerals represent chromosome numbers. Grey vertical lines separate chromosomes. Red denotes copy number increase, green copy number decrease. Genes are represented according to their position from left to right on each chromosome. Isolate 62 displays duplicated Chromosome I. The apparent subtelomeric amplifications are artifacts of DNA preparation. Note that BY4743 is a diploid strain, whereas all isolates are haploid.

Since starved cultures showed no significant increase in mutation frequency, but did exhibit a dramatic increase in chromosomal rearrangements, we tested their genomes for additional evidence of genomic restructuring by DNA array-Comparative Genomic Hybridization (aCGH). aCGH uncovers net changes in the relative amount (copy number) of genomic DNA regions [47]. We performed aCGH on four reproductively isolated starved isolates (61, 62, 65 and 68), two starved isolates that did not show decreased fertility in backcross (71 and 73), two unstarved control isolates (42 and 45), and the ancestral strain BY4743. Though all analyzed isolates exhibited differences in genome content spread throughout their genomes, starved isolate 62 was most distinctive in that it contained an entire extra copy of Chromosome I, i.e., it was disomic for this chromosome in an otherwise haploid background (**Fig. 3**). The aCGH finding that isolate 62 contains two copies of Chromosome I was confirmed by densitometry of a pulsed-field gel (**Fig. S1**).

### Whole genome sequencing reveals few sequence changes in isolate 62

To confirm Chromosome I disomy and to discover other possible changes in the genome of isolate 62, we performed Ion Torrent sequencing of its genome, using unstarved haploid ancestor BY4741 as a control. As expected, the coverage of Chromosome I in disomic isolate 62 was twice the average coverage for the genome, 42X vs. 21X, respectively, whereas in the unstarved control coverage was similar across all chromosomes. Indeed, isolate 62 revealed a 3.25-fold higher incidence of substitutions on Chromosome I (4.78×10^−5^ vs. 1.47×10^−5^ average in the 62 genome, p = 7.92*10^−7^, assuming Poisson-distributed mutation across the genome), further supporting the disomic nature of isolate 62. In terms of mutations that could affect sporulation, sequencing of isolate 62 revealed just one mutation with a potential sporulation defect, a G>A transition mutation in *UBP3*, encoding a ubiquitin protease (*ubp3-62*) [47]. A nonsense mutation in *UBP3* has been previously reported to mildly affect sporulation in a heterozygote and strongly affect sporulation in a homozygote [47]. However, because we found that the isolate 62 self-cross, homozygous for the *ubp3-62* missense mutation, undergoes meiosis at the wild type level (**Fig. 2**) and the heterozygous *ubp3-62/UBP3* backcross had low sporulation frequency, we concluded that the *ubp3-62* missense mutation in isolate 62 did not affect sporulation in a manner similar to the previously described nonsense *ubp3* mutation.

Finally, the sequenced genome of isolate 62 was checked for genomic rearrangements (breakpoints). All reads were mapped to the contigs containing a potential breakpoint and no structural variants were identified, aside from the extra copy of Chromosome I.

### Tetraploidization rescues meiosis

The presence of another copy of Chromosome I, coupled with the apparent absence of any fine-scale mutations that could explain the meiotic defect of starved isolate 62, suggested that the chromosomal duplication isolate 62 was the culprit for defective meiosis. If true, tetraploidization of the 62 backcross should rescue its meiotic defect, as, in tetraploid configuration, each copy of Chromosome I would have a homologue with which to pair [40]. Tetraploids of the low-fertility 62 backcross, along with its unstarved control, were generated via plasmid-generated *HO* expression and mating. Once tetraploidy was confirmed, the fertility of several independent tetraploid clones and their control (tetraploidized unstarved ancestor) were assayed for sporulation efficiency and spore viability.

**Figure 4:**
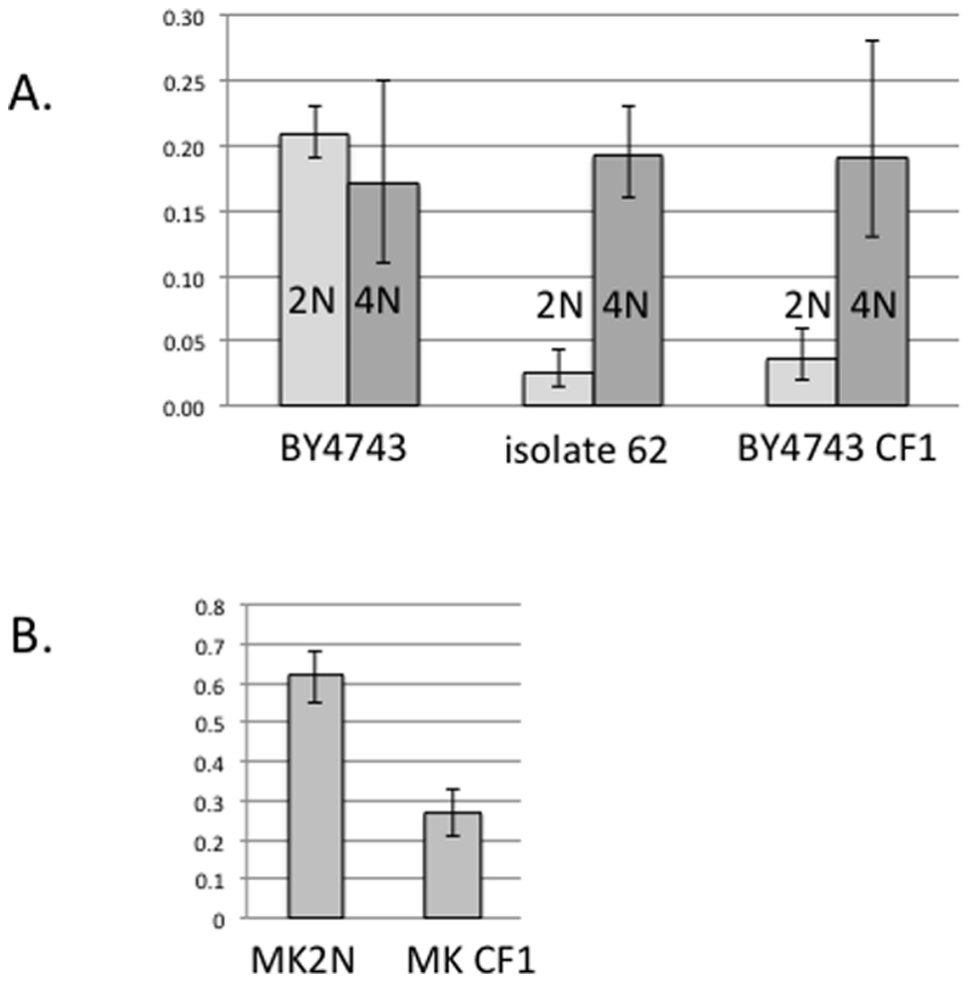
The sporulation defect of starved isolate 62a and chromosome fragment-containing strains is cured by tetraploidization. **A**. Light grey: sporulation frequencies of unstarved diploid control (BY4743), selfed starved isolate 62a and the unstarved BY4743 containing a Chromosome fragment of Chromosome I (CF1); dark grey: their tetraploid derivatives. **B**. Sporulation frequencies of a diploid SK1 derivative strain containing CF1 (MKCF1) and its control euploid (diploid) strain (MK2N). Error bars are 95% Wilson's binomial CI.

As expected, we found that tetraploidization had an insignificant effect (1.2 fold) on the sporulation frequency of the unstarved ancestor BY4743 (p = 0.52, Fisher's exact test) (**Fig. 4A**). In contrast, the tetraploidized isolate 62 backcross showed a 6.3-fold increase in sporulation frequency compared to the diploid backcross (p<0.0001, Fisher's exact test) (**Fig. 4A**). Additionally, starved isolate 62's tetraploid sporulation frequency was indistinguishable from that of the unstarved tetraploid. As expected, we found no significant changes in spore viability between the diploid isolate 62 backcross and its tetraploidized derivative (data not shown). The results from tetraploidization experiments, taken together with the results from forward mutagenesis and sequencing, confirm that the nature of the meiotic defect we observed in isolate 62 is chromosomal, rather than genic or sequence-based.

Interestingly, backcross tetraploidization of the three other isolates also restored their sporulation frequency (data not shown). Because a multitude of small copy number variations (CNV) exists in these isolates (**Fig. 3**), uncovering the basis for reproductive isolation in these other strains will require a systematic investigation of each CNV, singly and in different combinations. Here, we focused on further exploring the possibility that a major genome restructuring event, i.e., the accumulation of an extra copy of a chromosome, can reduce fertility in yeast.

### Chromosome fragment I reduces fertility in a naïve strain

If an extra copy of Chromosome I indeed reduces fertility in the starved isolate 62 backcross, then fertility of an unstarved (naïve) diploid strain bearing a supernumerary Chromosome I should also be lower. To investigate this possibility, we introduced a chromosome fragment bearing approximately 190 kb, or 82%, of Chromosome I (CF1) sequence into the diploid strain BY4743 (named 4743CF1). After confirming the presence of the fragment using PFGE (data not shown), we compared sporulation frequency and spore viability of strain 4743CF1 with those of the ancestral diploid and the starved isolate 62 backcross. Sporulation frequency of strain 4743CF1 was significantly lower than that of the diploid BY4743 (p<0.0001, Fisher's exact test) and indistinguishable from that of the starved isolate 62 backcross (**Fig. 4A**).

Interestingly, spore viability was somewhat lower in strain 4743CF1 compared to BY4743 (75% vs. 93%). However, these values had overlapping 95% binomial confidence intervals and the Fisher's exact test showed borderline association between having the chromosome fragment and decreased spore viability in three independent replicates. We observed similar differences in spore viabilities in the initial starved isolates, however, the association between starvation and spore viability was not significant after multiple comparison correction at 95% confidence (data not shown).

In addition to using BY4743, a low-sporulating S288c diploid derivative used here because it did not sporulate during the starvation experiments (See Materials and Methods), we examined the effects of introducing an extra chromosome fragment into a strain that has high sporulation frequency. We used MK001, a *ura3* haploid derivative of the high-sporulating SK1 strain (kind gift of A. Kirchmaier), diploidized it (MK2N), introduced CF1 as described above, and sporulated the resulting strain MKCF1. The chromosome fragment-containing MKCF1 sporulated at 27% vs. diploid MK2N at 62%, a significant difference (p<0.0001, Fisher's exact test) (**Fig. 4B**). As with BY4743, spore viability was not significantly different between diploid MK2N and MKCF1 strains (data not shown). These experiments demonstrate that the presence of an extra fragment of Chromosome I reduces yeast fertility in two different *S. cerevisiae* backgrounds.

### Validation of tetraploidization test in CF1 containing strains

Our tetraploidization experiments were undertaken to help distinguish between a chromosomal and a genic basis for post-zygotic reproductive isolation. To demonstrate that the tetraploidization test works in yeast stains that contain no changes except an extra homologous chromosome, we tetraploidized the chromosome fragment-containing 4743CF1 and measured its sporulation frequency in relation to the tetraploid BY4743 derivative without CF1. Tetraploidization restored sporulation frequency to that of the non-CF1 containing tetraploid (p<0.0001, Fisher's exact test), albeit with a somewhat increased margin of error (**Fig. 4A**).

This result confirms the validity of the tetraploidization test in yeast as the chromosome fragment was the only genetic difference between the BY4743 and 4743CF1 strains.

### Other supernumerary chromosomes inhibit meiosis

To determine whether the addition of chromosomes other than Chromosome I or its fragment could influence meiosis we used diploid strains that contain other supernumerary chromosomes. To this end we employed four haploid strains that contained one or several extra Chromosomes – one with an extra Chromosome IV, one with an extra Chromosome VI, one with two extra Chromosomes, VI and XII, and another with three extra Chromosomes, II, VI and XII (kind gift of K. Anders) [48] – and crossed each to congenic haploid strain BY4742. The resulting four single or multiple trisomic diploid strains were sporulated, and their sporulation efficiency and spore viability were measured.

**Figure 5:**
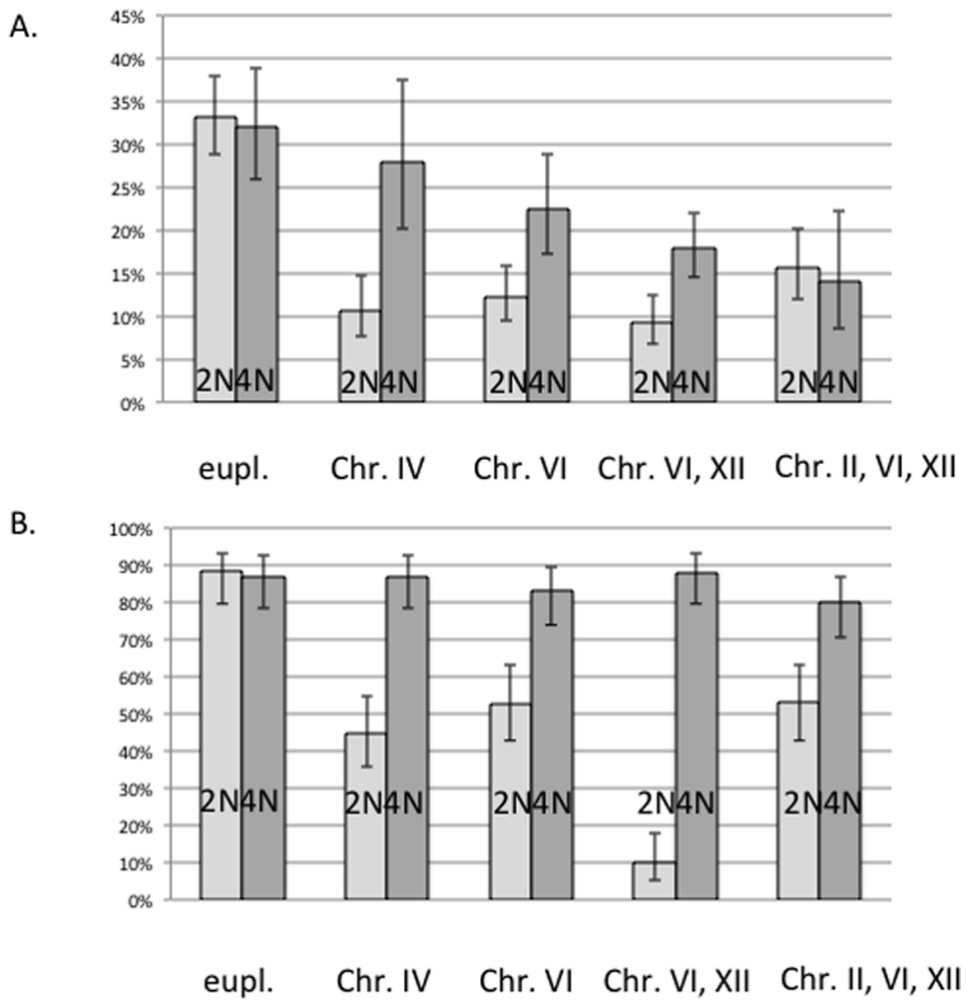
Extra chromosomes decrease (A) sporulation frequency and (B) spore viability, which are cured by tetraploidization to different extents (see text). Light grey, 2N – diploid strain and its derivatives containing one or several supernumerary chromosomes, as indicated. Dark grey, 4N – tetraploid derivatives. Error bars are 95% Wilson's binomial CI.

Diploid strains bearing one or several extra chromosomes also exhibited lower sporulation frequencies (in all trisomics vs. diploid control, p<0.0005, Bonferroni-corrected Fisher's exact test) (**Fig. 5A**). Interestingly, spore viability in the diploid trisomics ranged from 10% to 53%, which was markedly lower than that of the euploid control strain, a statistically significant association (p<0.01 for all four cases, Bonferroni-corrected Fisher's exact test) (**Fig. 5B**). These experimental results suggest that several unbalanced chromosomes besides Chromosome I can disrupt meiosis in diploid trisomics.

### Tetraploidization of trisomics partially restores fertility

To determine whether changes in fertility in diploid trisomics could be attributed to supernumerary chromosomes, we tetraploidized the trisomic strains and scored their sporulation efficiency and spore viability. In cases of single trisomics for Chr. IV, Chr. VI and the double trisomic for Chrs. VI and XII, tetraploidization restored the sporulation frequency, (diploid vs. tetraploid, extra Chr. IV p = 0.002, extra Chr. VI, p = 0.031, extra Chrs. VI and XII, p = 0.048, one-tailed Fisher's exact test), albeit to a level marginally lower than that of tetraploidized BY4743 (**Fig. 5A**). However, in the case of the triple trisomic (Chrs. II, IV and XII), sporulation frequency of the tetraploid was not restored (diploid vs. tetraploid p = 0.75, one-tailed Fisher's exact test). Altogether, these data indicate that multiple supernumerary chromosomes may bring about gene dosage effects on sporulation frequency that cannot be cured by tetraploidization.

Finally, regarding spore viability, in all four cases of single and multiple extra chromosomes, tetraploid spore viability was restored to levels indistinguishable from that of the balanced diploid (Fisher's exact test, all cases p>0.6) (**Fig. 5B**). Thus, we observed no gene dosage effects on spore viability in strains containing extra chromosomes.

## Discussion

Starvation can be viewed as an outcome of any number of severe environmental challenges that species invariably face at some point in their life histories [9], [49]. We used this treatment to investigate the effects of severe stress on panmixis. We found that starvation in yeast produces a burst of genomic restructuring that impacts not only asexual but also sexual reproductive capacity, in some cases leading to post-zygotic reproductive isolation.

We assayed 80 clones from starved cultures for their fertility and 271 clones from starved cultures for chromosomal rearrangements. Since PFGE analysis detects only some chromosomal rearrangements, we refrained from directly comparing fertility and the apparent presence (or lack) of rearrangements. Rather, we concentrated on studying rearrangements in the randomly chosen meiotic products from starved cultures, using genetic methods. Thereby we showed that prolonged starvation diminishes survivors' fertility and the fertility of their backcrosses by diminishing their gametic output. Genetic manipulation, deep sequencing and array-comparative genomic hybridization independently confirm that this effect can arise from genomic, rather than genic, changes. Diminished fertility arises from decreased sporulation frequency, as the viability of rare spores produced by starved diploids was generally high, and not significantly different from that of the unstarved diploid control.

Accumulation of supernumerary chromosomes is a frequent genomic response to stressful environments [50], [51]. We found that the presence of a single or several supernumerary chromosomes tends to lower sporulation frequency and, in the case of multiple supernumerary chromosomes also affects spore viability. We suggest that a single extra chromosome impacts meiosis via presentation of an extra homologue, which may disrupt synaptonemal complex formation for this chromosome [18]–[20]. In our experiments, the genic effects on meiosis, presumably arising from either expression imbalance or epistasis, appear only when there is more than one extra chromosome. We have thus found that while an extra homologous chromosome fragment or one or several supernumerary chromosomes diminishes yeast fertility, tetraploidization restores fertility in these strains to wild type levels, validating, for yeast, a prediction made 80 years ago by Dobzhansky [40].

### A role for genomic restructuring in reproductive isolation

Although we observed a remarkable increase in the number of genomic rearrangements in clones surviving starvation, we did not observe a concomitant increase in fine-scale mutations, judged both by genetic assays and by deep sequencing. These observations are consistent with a similar finding in *Candida albicans*, where several different stressful environments induced a recombination-driven loss of heterozygosity, but not a concomitant increase in the rate of point mutations [4]. Could it be that eukaryotic genomes evolutionarily adapt more quickly to stress via genome restructuring than by sequential accumulation of point mutations? Stress-driven mutation has been extensively documented in prokaryotes [35], [52], [53], and in these taxa small-scale genetic changes can play important roles in adaptive evolution. By contrast, eukaryotic cells with their relatively higher gene densities and longer generation times may be better equipped to respond quickly to stress via genomic restructuring. Indeed, evolutionary change via combinatorial reassembly of existing loci has been proposed to underlie abrupt phenotypic changes in plants and in metazoans [54]–[56]. A related phenomenon, aptly termed “chromosome catastrophe” [57] is well-known during carcinogenesis. Stress-induced genomic restructuring has been demonstrated to occur in every eukaryotic Kingdom in response to high temperature, high salinity, partial desiccation and limiting nutrients [3], [5], [33], [55]. In yeast, genomic rearrangements and copy-number variants arise in response to chemical treatment [58], as well as to nutrient limitation [5], [6], [59], [60] and depletion [4], [9]. In short, the widespread occurrence of genomic restructuring under stress provides ample opportunities for rapid reproductive isolation.

### Alternative models of post-zygotic reproductive isolation

Three genetic models have been proposed to explain post-zygotic reproductive isolation in yeast: genic, chromosomal, and anti-recombinational [28]. To date, hybridization and reconstruction experiments have failed to rule out any of these possibilities, and indeed, genomic rearrangements [61], the anti-recombinational function of meiotic mismatch repair [43] and epistasis [62] may all play a role in interspecific hybrid sterility. While elegant experiments [28] have recently shed light on which genetic factors separate extant yeast species, none definitively reveal the molecular basis of the *initial* event that created a reproductive barrier. Here, we have shown that partial reproductive isolation (low hybrid fertility) can arise from large-scale genomic restructuring, rather than from fine-scale mutational causes (e.g., sequence divergence and epistasis). Our study does not directly distinguish between chromosomal and mismatch repair anti-recombination-driven models. However, given that the level of sequence divergence was very low in our experiments, certainly in relation to what has been found to activate yeast meiotic mismatch repair [63], we believe that the mismatch repair mechanism is unlikely to explain our observations of reproductive isolation. We suggest that mismatch repair anti-recombination helps to enforce reproductive isolation between extant species once their sequences have diverged enough to activate this mechanism [43].

Our observations that diminished sporulation frequency (but not diminished spore viability) may drive reproductive isolation agrees with the only other experimental study of incipient speciation in yeast, which was promoted by divergent selection [33]. The findings of our study and that of Dettman et al. differ from observations made by crossing extant members of the *Saccharomyces* genus group [24], [28], [62], [64], where spore viability was found to be extremely low. Interestingly, crosses between diverged wine strains of (apparently) *Saccharomyces cerevisiae* have revealed the full gamut of changes in both spore viabilities and sporulation frequencies [25]. However, these assays were also performed on extant lineages having varied evolutionary histories and not on strains experimentally evolved from a single clone. We suggest that genic and genomic changes that accumulated after these lineages diverged result in decreased hybrid spore viability; these changes shed little light on the initial mechanism of speciation.

### Implications for sympatric speciation

Many studies have shown how divergent selection can lead to the evolution of reproductive isolation in allopatry [16], [41]. This mechanism requires geographic isolation and many generations, which enable populations to accumulate enough genetic differences, be they genic or chromosomal, to create a reproductive barrier. We have described how a single chromosomal change can instantaneously create a barrier to panmixis in sympatry. We propose that nascent speciation events, driven by aneuploidy in environmentally stressed populations [9], [50], [51], may arise frequently, but these new variants only rarely become fully-fledged species. To do so, they must overcome genetic drift, and contain traits that are beneficial in the stressful environment and also not impose a significant cost in environments optimally suited for yeast reproduction. To note, natural and industrial *Saccharomyces* isolates are frequently aneuploid [65]–[67], perhaps representing the genomes that diverge away from the “canonical” *Saccharomyces* chromosomal arrangement.

### A meiotic monkey wrench

We have found that a single extra homologous chromosome greatly reduces gametic output in yeast. Similar phenomena have been described in Down syndrome patients, where abortive meioses occur in unbalanced germ lines, with observable trivalents during meiotic prophase [68], as well as in murine meiosis, where Robertsonian translocations strongly correlate with impaired spermatogenesis [20]. In yeast, proper (bivalent) synaptonemal complexes are monitored by the pachytene checkpoint, which can abort meiosis if synaptonemal complexes are improperly formed [22], as well as by the meiotic spindle checkpoint that prevents meiotic progression in response to the presence of unsynapsed chromosomes [69]. These checkpoints can bring about low sporulation, the very effect we see in our studies.

### Speciation in stressful conditions may itself be adaptive

Stressed populations can rapidly become polymorphic, and in certain instances the bulk of genomic changes is confined to a small subset of the stressed population [52], [70]. The capacity to undergo stress-associated genome restructuring, some of which may lead to reproductive isolation, could be indirectly advantageous in a novel or changing environment. If a restructured genome contains a specific set of alleles that is beneficial in the novel environment, then mating and recombination with less fit members of the population should be disfavored [71]. Restricting gene flow would therefore benefit the new adapted clade, providing a molecular basis for subsequent reinforcement [72], [73]. It is therefore tempting to speculate that in heterogonic species such as yeast reproductive isolation may itself be indirectly adaptive, especially in the times of stress.

## Materials and Methods

### Strains and culture conditions

We employed congenic laboratory strains BY4741 (*MAT a his3Δ01 leu2Δ0 met15Δ0 ura3Δ0*), BY4742 (*MAT alpha his3Δ01 leu2Δ0 lys2D0 ura3Δ0*), their diploid hybrid BY4743, s288c derivatives [32] and MK001, a *ura3* haploid derivative of the high-sporulating SK1 strain (kind gift of A. Kirchmaier) (Table S1). We used a low-sporulating s288c strain in the initial starvation experiments because the strains of other genetic *S. cerevisiae* backgrounds sporulate when starved. Haploid disomic strains KAY638, KAY605, KAY679, KAY681 and their ancestral strain KAY600 congenic to BY4741 were a kind gift of K. Anders. Starvation treatment, karyotyping and forward mutagenesis assays were performed as previously described [9].

### Genetic manipulations

#### a. Generation of chromosome fragment CF1

A 190 kb chromosome fragment was generated using the procedure described in Morrow et al. [74]. For this project, a 364 bp DNA sequence from Chromosome I (position 146139 to 146537) was ligated into a fragmentation vector pYCF4 (kind gift of P. Hieter) to make fragmentation vector CFV1. Linearized CFV1 was transformed into diploid unstarved yeast to make chromosome fragment CF1.

#### b. Self-crosses/Tetraploidization

To switch the mating type, cells were separately transformed with plasmids p37HO or p38HO, which bear genes for hygromycin and G418 resistance, respectively, and contain HO endonuclease under control of the GAL1/10 promoter (kind gift of K. Schwartz). Each HO transformant pair was pre-grown in YEP raffinose (20 g L^−1^), and HO expression was induced by adding galactose (20 g L^−1^) to each culture. Each strain was then incubated (with agitation, to prevent mating) overnight at 30°C. Following induction, each strain pair was mixed and allowed to mate for 4–6 h, following which cells were diluted and plated to selective media containing 200 µg ml-1 G418 and 300 µg ml-1 hygromycin. Only zygotes carry resistance to both antibiotics and grow on double antibiotic plates. Depending upon whether haploid starved isolates or diploid hybrids underwent this procedure, either diploid self-crosses or tetraploids were obtained, respectively. Before analysis both plasmids were segregated out by serial passage on rich medium. Ploidy was confirmed by mating tests, microscopic observation and/or FACS analysis.

#### c. Sporulation and analysis of sporulation

Sporulation was initiated by inoculation into 1 mL sporulation medium (1% potassium acetate, 0.1%yeast extract, 0.05% glucose and auxotrophic supplements) and incubating at 25°C for 72 h, in the case of S288c-derived strains, and for 24 h, in the case of SK1 and its derivatives. Sporulation was assessed microscopically at 400× magnification using phase contrast. Sporulation frequency was estimated as the ratio of asci (sporulated cells) to the total number of cells. Spore viability was calculated as the ratio of germinated spores (microcolonies after 8 h on solid rich medium, 100× magnification) versus total number of spore bodies, following random spore analysis [75].

### Array-Comparative Genomic Hybridization

Two-color array-Comparative Genomic Hybridization was performed by using microarrays with PCR products corresponding to full-length ORFs from the S288C strain of S. cerevisiae. Target DNA was isolated from starved isolates and from the diploid ancestral strain BY4743, and then individually labeled with Cy5 dye (red). Likewise, genomic DNA isolated from strain S288c (closely related to BY4743)was labeled with Cy3 dye (green) to serve as the reference signal for each spot. For each isolate, its Cy5-labeled DNA was mixed with an equimolar amount of the S288c Cy3-labeled DNA and the mixture hybridized to the microarrays. Array hybridization conditions, washing, scanning, and data collection were all performed as described in Dunn et al. 2005 [76]. Experiments were performed in duplicate for all strains. We collected data for all spots with robust hybridization to the reference S288c DNA, which we defined as those where the ratio of Cy3 mean signal intensity to Cy3 median background intensity was >2.0. We did not filter the data based on any measurements of Cy5 intensity, so that we could detect any regions that had been deleted (see top portion of Table S2 for data filtering parameters). The data are displayed in Fig. 3 and in Table S2 as the log2 of the averaged Red/Green (Cy5/Cy3) normalized ratios (mean) from each gene's two technical replicate spots.

### Ion Torrent Sequencing

Yeast DNA was prepared using standard methods [77]. Fragment libraries were prepared for sequencing on the Ion Torrent Personal Genome Machine (PGM) using the standard Ion Torrent fragment library kit following the manufacturer's instructions (Life Technologies). The 314 and 316 chips were used to sequence the genomic DNA libraries using the PGM Sequencer [78]. CLC Genomics Workbench (4.7.2) software package was used to perform the mapping and visualization. PGM reads were also assembled de novo to search for structural variation in the genomes usingthe CLC Genomics Workbench as well as Velvet [79]. Bowtie [80] was used to map the PGM reads for the Velvet reference guided de novo assembly. The raw assembly included 10.8×10^6^ bases with a maximum contig of 49,938 bp and an N50 of 11,369. All contigs from CLC Genomics Workbench (4.7.2) and Velvet were aligned to the s288c genome from NCBI (NC_001133-NC_001148 and NC_001224). All alignments were analyzed to search for potential breakpoints. Reads from both 62 and BY4741 were then mapped to all contigs containing a potential breakpoint. Sequence data have been uploaded to the Sequence Read Archive (http://trace.ncbi.nlm.nih.gov/Traces/sra/) under accession numbers SRA050328 and SRA050381 for 62 and BY4741, respectively.

### Statistical tests

The Kruskal-Wallis and Mann-Whitney U tests were employed for comparing samples while sampling the divergent and thus, *a priori*, non-normal, starved populations. Wilson's binomial confidence intervals and Fisher's exact test for association was used when comparing clonal populations' binary data, such as sporulation frequency or spore viability. Except where noted tests were run as two-tailed, at 95% confidence. Statistical tests were performed using the R platform using packages approved by the R Development Core Team.

## Supporting Information

Figure S1 **Pulsed-field gel electrophoresis reveals that Chromosome I is duplicated in isolate 62.** The PFGE parameters were adjusted to separate smaller chromosomes. Chromosome I is the smallest chromosome in the yeast genome. M – Yeast Chromosome PFG marker (New England Biolabs), A – unstarved haploid strain BY4741, 62 – starved isolate 62. Red arrow denotes the duplicated chromosome.
(TIFF)

Table S1 **Ancestral strains used in this study.**
(PDF)

Table S2 **Normalized log(2)-ratios of Red/Green signals on spotted DNA arrays (see** **Fig. 3** **).**
(XLSX)
