# Supplementary figures and images for: Starvation-Associated Genome Restructuring Can Lead to Reproductive Isolation in Yeast
Source: PLoS One. 2013 Jul 24;8(7):e66414. doi: 10.1371/journal.pone.0066414 (PMC3722211; doi:10.1371/journal.pone.0066414)

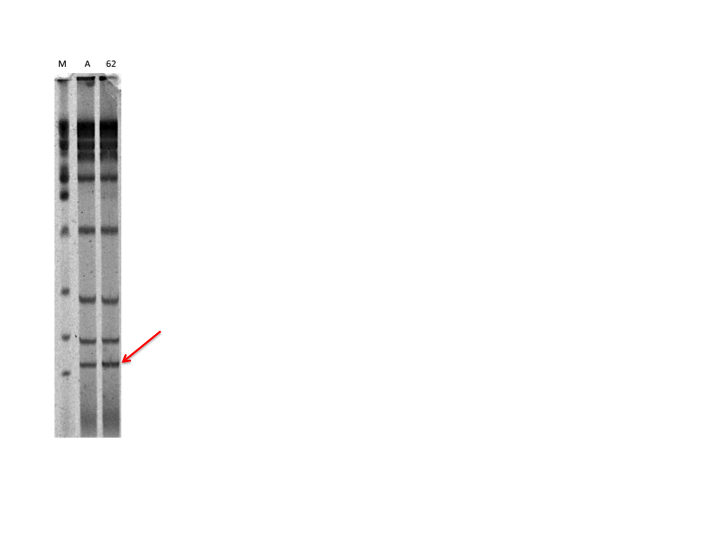

Supplement: Figure S1 — Pulsed-field gel electrophoresis reveals that Chromosome I is duplicated in isolate 62. The PFGE parameters were adjusted to separate smaller chromosomes. Chromosome I is the smallest chromosome in the yeast genome. M – Yeast Chromosome PFG marker (New England Biolabs), A – unstarved haploid strain BY4741, 62 – starved isolate 62. Red arrow denotes the duplicated chromosome. (TIFF) [file pone.0066414.s001.tiff]
