# Supplementary material for: Starvation-Associated Genome Restructuring Can Lead to Reproductive Isolation in Yeast
Source: PLoS One. 2013 Jul 24;8(7):e66414. doi: 10.1371/journal.pone.0066414 (PMC3722211; doi:10.1371/journal.pone.0066414)
Supplement: Table S1 — Ancestral strains used in this study. (PDF) [file pone.0066414.s002.pdf]

Strain	Genotype	Reference
BY4741	*MATa his3Δ1 leu2Δ0 met15Δ0 ura3Δ0*	[33]
BY4742	*MATalpha his3Δ1 leu2Δ0 lys2Δ0 ura3Δ0*	[33]
BY4743	*MATa/alpha his3Δ1/his3Δ1 leu2Δ0/leu2Δ0 met15Δ0/MET lys2Δ0/LYS ura3Δ0/ura3Δ0*	[33]
MK001	*MATa cyh2 ho::LYS2 leu2::hisG lys2 trp1::hisG ura3-1*	A. Kirchmaier
KAY 614	*MAT*a *his3-Δ200 ura3Δ0 cen4-458::*P_{GAL1}*-CEN3 ura3::HIS3(at CEN4)*	[48]
KAY 600	*MAT*a *his3-Δ200 leu2Δ0 ura3Δ0 cen6-483::*P_{GAL1}*-CEN3 ura3::HIS3(at CEN6)* [pRB327 *TUB1 LEU2 2-micron*]	[48]
